# Increasing Physical Activity in Medium Secure Mental Health Services in the UK: (IMPACT) - Preliminary Results from the Phase 4 Feasibility Study, with a highlight into the Women’s Services

**DOI:** 10.1192/j.eurpsy.2024.233

**Published:** 2024-08-27

**Authors:** T. Walker

**Affiliations:** Psychology, Durham University, Durham, United Kingdom

## Abstract

**Introduction:**

In the UK there are 3500 individuals detained in medium secure forensic psychiatry units. Service users in such settings have complex and serious mental illness (SMI), often with co-morbid physical health problems and a life expectancy of at least 10 years shorter than the general population. They often have low levels of physical activity. There is little evidence about physical activity interventions for medium secure service users in the United Kingdom.

**Objectives:**

Our objective is to co-produce, with medium secure service users, the content and delivery of an intervention to increase physical activity. We shall assess feasibility, acceptability, and pilot data collection methods for outcomes relevant for a future randomised controlled trial.

**Methods:**

This is a 30-month mixed-methods project that will follow the Medical Research Council (MRC) framework Developing and Evaluating Complex Interventions. The study has 4 phases. Phases 1-2 will gather information required to co-develop an evidence-based intervention in Phase 3. Phase 4 will assess the intervention in a feasibility study, evaluating and testing the intervention for a future pilot study.

Study settings: Two NHS Medium Secure In-Patient Psychiatric Hospitals in the UK.

**Results:**

This paper presents the preliminary findings from Phase 4 and also offers a highlight into the results from the Women’s Services from both study sites. A total of thirty-three service users from both study sites participated in Phase 4 of the study and twenty-six completed the physical activity intervention, known as the IMPACT Intervention. Between both study sites, there were two Women’s Standard Medium Secure Services and one Women’s Enhanced Medium Secure Service, involved in this study. A total of nine female service users participated in Phase 4.

**Conclusions:**

The preliminary findings of Phases 4 are allowing the team to move forward and evaluate the effect of the IMPACT Intervention.

**Disclosure of Interest:**

None Declared

